# Proceedings of the Buffalo Medical and Surgical Association

**Published:** 1888-12

**Authors:** Wm. A. Hoddick

**Affiliations:** Secretary pro tem.


### PROCEEDINGS OF THE BUFFALO MEDICAL AND SURGICAL ASSOCIATION.

Reported by WM. A. HODDICK, M. D., Secretary *pro tem.*

Regular monthly meeting of the Buffalo Medical and Surgical Association, held in the Mechanics' Institute Building, on Thursday, Nov. 8th, at 8.30 P. M.

The President, Dr. P. W. VanPeyma, in the chair.

Present: Drs. Lothrop, Tremaine, Strong, Hebenstreit, Falk, W. W. Potter, Burwell, Hoddick, and Hubbell.

Minutes read and approved.

The name of Dr. Rogers was presented for membership.

Dr. Thos. Lothrop presented the following memorial:

### IN MEMORIAM.

### Fred. R. Campbell, A. M., M. D.

Died September 14, 1888, aged 28 years and 7 months.

A life full of promise and of usefulness to the world and to the profession, has been brought to a close in the early death of the subject of this notice. Encomiums of deserved praise have been lavished upon his brief and remarkable career. Coming to this city a stranger, and without social or financial support, which render advancement in life an easy task, he overcame all obstacles to success by his industry, and advanced step by step until, at a very early age, he was recognized as one of the most highly accomplished of the junior members of the profession in our city, as a fine classical scholar and writer, as a successful medical teacher, and as a genial and honorable associate and friend. Patient industry and unremitting labor were the secrets of his success.

We beg to refer to his personal qualities, which commended him to the members of this Association. Without any self-assertion, and with a modesty of demeanor which made him always the agreeable associate, he commanded the love and respect of all and was the center of a circle of warm and devoted friends, who loved him for the purity of his life, the constancy of his affections, and the brilliancy of his intellectual attainments.

We refer with pride to his literary efforts, by which he bequeathed to the profession his only published work, "The Language of Medicine," which has received, through the medical press, the most favorable criticisms for its intrinsic worth.

While yet young, he was called to a most important chair of the Medical Department of Niagara University, which he filled with signal ability, and gave promise of a capacity to occupy a leading position in the faculty of that growing school of medicine.

In the social and medical organizations with which he was identified, the products of his pen exhibited the result of patient and laborious study, and extensive medical, classical and philological learning.

The death of one so well endowed by nature, and so thoroughly equipped by education for the work of life, in the special field of scientific and philanthropic labor which he had chosen, is a loss beyond these feeble words to express, and calls forth our sympathy and our sorrow. We lament his early taking off, for the profession loses a bright intellect, a pure heart, a noble nature. We believe the loss we sustain receives its merited compensation in the gain to him of rest in the world beyond, where with him it will be

> "Better yet and better still
> In infinite progression."

Dr. W. W. Potter moved that the memorial be spread upon the minutes of the Association, as a record of respect for the deceased, that it be published in the local medical journals, and a copy of same be sent to family. Some discussion followed as to the propriety of having the memorial printed in the secular press. The motion, as given, was finally adopted.

Remarks in eulogy of the late Dr. F. R. Campbell were also made by Drs. Potter, Strong, Tremaine, Lothrop, and others, all sincerely regretting that a life so full of promise had been so suddenly cut off.

VOLUNTARY CONTRIBUTIONS.

DR. VANPEYMA presented two clinical reports on obstetrics. The first, illustrating the use of small doses of ergot (five drops of ext. fld. every fifteen or twenty minutes), as recommended by Dr. Stockton: Three weeks ago he was called to a case where the membranes had ruptured and all contractions had ceased. Small doses of ergot stimulated contractions, followed by a normal delivery. The second, a midwife case, arm and part of head presenting. He succeeded in bringing the head into the superior strait, and delivered.

DR. LOTHROP thought the second case one in which cephalic version, by pushing up the shoulder and the arm presenting, and pushing the head into the pelvis with the hand on the abdomen, could have been performed.

As to the first case reported, he was decidely averse to using ergot at any time before the expulsion of the fetus, or even before the delivery of the secundines. He believed it did more harm than good, excepting where contractions could not be otherwise excited. During the past ten years he has used it but twice.

DR. STRONG was surprised at Dr. Lothrop's infrequent use of ergot. He found it indispensable in about one-fifth of his cases.

DR. WHEELER was then invited to present his paper, subject, "Certain Diseases of the Testicle." He confined himself almost entirely to the subject of hydrocele, giving a complete résumé of the anatomy, pathology and etiology of the parts affected. In treatment he favored the method by incision of the tunica vaginalis, allowing obliteration of the sac to take place by granulation, under an antiseptic dressing.

DISCUSSION.

DR. TREMAINE could not permit so admirable a paper to pass without some notice. Essayists of the present day generally strive to present something new, which is often of little real value. Dr. Wheeler had presented an old subject in a very concise and complete manner. In treating hydrocele, he injected carbolic acid. He used it pure, injecting about one-half of a drachm into the sac, kneading and squeezing the most of it out again. In his hands the treatment had been universally satisfactory. Iodine injections were often successful, the result being frequently attained without destruction of the sac. Sometimes he made an incision, sewing the two layers of the sac together with a few strands of catgut.

Dr. Wheeler, in closing the discussion, said that his experience with carbolic acid had been unsatisfactory. He had tried the Levis method, injecting from ten to twenty drops of carbolic acid. It often caused great pain, and failed to cure in ten per cent. of the cases. He thought it better practice to incise the scrotum, drain the sac, and allow it to fill up with granulations, under an antiseptic dressing.

Dr. Tremaine thought it unnecessary to drain the sac, as aglutination of the two tunics would take place after incision. He never knew of a case that did not get well after an incision had been made.

MISCELLANEOUS BUSINESS.

Essayists for the next meeting were announced: Dr. F. H. Potter, subject, "Treatment of Acute Coryza;" Dr. Roswell Park, subject, "Radical Cure of Hernia."

Adjourned to the first Tuesday in December.

